# Dietary lipids induce PPARd and BCL6 to repress macrophage IL-23 induction after intestinal injury and LPS exposure

**DOI:** 10.21203/rs.3.rs-6557500/v1

**Published:** 2025-05-29

**Authors:** Ashleigh M. Gil, Daniel F. Zegarra-Ruiz, Wan-Jung Wu, Kendra Norwood, Adrien Assie, Buck S. Samuel, Angela M. Major, Gretchen E. Diehl, Andrea A. Hill McAlester

**Affiliations:** Baylor College of Medicine; University of Virginia; Baylor College of Medicine; Baylor College of Medicine; Baylor College of Medicine; Baylor College of Medicine; Texas Children’s Hospital; Memorial Sloan Kettering Cancer Center; Baylor College of Medicine

## Abstract

Unresolved tissue damage is a common feature of Inflammatory Bowel Disease (IBD) that facilitates disease progression. Here, we showed that high animal fat diets (HFD), an environmental risk factor associated with IBD pathogenesis, suppress intestinal macrophage production of critical tissue repair responses after damage. This includes reduced IL-23 production, which drives downstream production of the IL-22, which is needed for barrier repair. Indicating that dietary lipids interfere with responses to microbial molecules needed to induce barrier protective functions, we found oleic acid could directly suppress macrophage *Il23a* induction after lipopolysaccharide (LPS) treatment. Deleting the lipid transporter CD36 on macrophages restored the *Il23a* and *Il22*response, reducing intestinal damage in HFD-fed DSS-treated mice. We found that CD36-mediated intracellular lipid accumulation, mainly oleic acid, in macrophages leads to peroxisome proliferator-activated receptor delta (PPARd)release of the transcriptional repressor protein B-cell lymphoma 6 (BCL6). BCL6 suppresses *Il23a* transcription in microbe-exposed macrophages. The studies suggest dietary lipid modulation of the macrophage PPARd/BCL6 transcriptional repressor complex is a key mechanism of fat-associated defects in intestinal damage repair and immune dysregulation. Overall, our findings provide new insights into dietary lipid contribution to intestinal disease progression and identify new potential therapeutic targets to decrease diet-associated risk for IBD.

## Introduction

Excess consumption of diets high in animal fat causes intestinal mucosal barrier dysfunction and is associated with an increased risk for the development and pathogenesis of inflammatory bowel disease (IBD). However, how animal fat-rich diets drive risk for IBD development and contribute to IBD pathogenesis is incompletely understood. Most evidence highlights that animal-sourced high-fat diets (HFDs) drive IBD progression by inducing changes in microbial composition and epithelial damage that elicit tissue-damaging immune responses. However, little is known about the direct effects of the diet on immune functions that may influence IBD pathogenesis.

Disease progression in IBD can be partly attributed to inefficient repair of the damaged intestinal barrier. Repair after intestinal injury heavily relies on intestinal immune cell production of antimicrobial and reparative cytokines ^1–5^. Macrophages are among the first immune cells to respond to damaged tissue sites, including the intestine. Macrophage recognition of damage signals, including microbial products, and appropriate corresponding cytokine responses are critical to repairing the damaged intestinal epithelial barrier ^1–5^. Loss of the appropriate macrophage cytokine responses to intestinal damage can result in defective tissue repair ^1–5^. Highlighting the influence of diet on immune cell function in intestinal repair, we previously demonstrated that lipids found in the HFD directly impair macrophage clearance of apoptotic neutrophils, a key inducer of macrophage IL-10 production needed to support repair ^2,6^. While this study links diet with defective macrophage tissue repair functions, we did not address if HFD exposure impacted additional macrophage reparative functions, further limiting intestinal damage repair.

Microbial breach of the intestinal epithelium is a key damage signal that induces macrophage antimicrobial and barrier repair cytokine responses. Among these responses is macrophage production of IL-23, which induces IL-22 production by T cells and innate lymphoid cells to support microbial clearance and epithelial wound closure ^4,7,8^. Alterations of the IL-23/IL-22 pathways are associated with IBD pathogenesis ^9^. Additional microbial-induced macrophage cytokines, TNF and IL-10, play a key role in microbial clearance and barrier repair, and dysregulation of these signals is also associated with intestinal damage and IBD pathogenesis. It is unclear whether the diet directly influences macrophage production of antimicrobial reparative cytokine responses during intestinal injury and the resulting impact on intestinal healing.

In this study, we used acute HFD feeding in a dextran sodium sulfate (DSS) mouse model of colitis in combination with in vitro lipid treatment assays in bone-marrow-derived macrophage (BMDM) cultures to define the direct impact of dietary lipids on macrophage antimicrobial cytokine responses needed to support intestinal damage repair. We show that macrophage *Il23a* and downstream induction of *Il22* are lost in the cecum of HFD-fed mice with intestinal injury induced by DSS treatment. Using in vitro studies, we identified that the unsaturated dietary lipid oleic acid, the primary lipid in the HFD, suppressed macrophage expression and production of *Il23a* in response to LPS. This effect also extended to LPS-induced cytokines *Tnf* and *IlT10*. In vitro, lipid and LPS treatment revealed that intracellular accumulation of oleic acid and increased expression of the lipid receptor and transporter CD36 in macrophages corresponded with decreased *Il23a*, *Tnf*, and *IlT10* responses to LPS. We further find that macrophage-specific deletion of CD36 attenuated intestinal injury and restored *Il23a*, *Il22*, *Tnf*, and *Il10* responses in HFD-fed DSS-treated mice. Analysis of pathways downstream of CD36-mediated lipid transport revealed a role for an intracellular lipid sensor/transcriptional repressor complex formed by PPARd and BCL6 in inhibiting macrophage cytokine production. In vitro inhibition of PPARd or BCL6 restored *Il23a*, but not *Tnf* and *Il10*, expression in oleic acid and LPS-treated macrophages, highlighting oleic acid in contrast palmitic acid had no impact on macrophage *Il23a*, *Tnf*, and *Il10* response to LPS, demonstrating lipid-specific influences on macrophage responses to microbial signals. Collectively, our studies reveal that, during HFD feeding, lipid accrual in macrophages leads to loss of the IL-23-IL-22 response after intestinal damage due to induction of the PPARd/BCL6 transcriptional repressive complex, supporting defective intestinal damage repair.

## Results

### Intestinal IL-23 and IL-22 reparative responses are lost in HFD-fed DSS-treated mice

**Figure 1:**
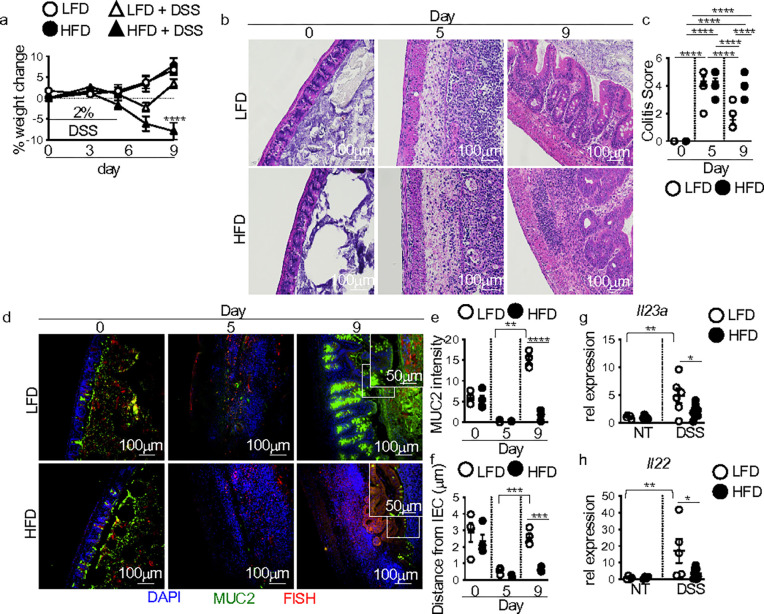
IL-23 and IL-22 responses are lost in HFD mice with unresolved intestinal damage. C57BL/6 mice were fed LFD or HFD for one week. Mice were then left untreated or treated with 2% DSS in the drinking water for 5 days. (**a**) Body weight (n = 8 mice/group). The following panels are measurements in the cecum of mice in panel (**a**). (**b**) Representative H&E staining and (**c**) blinded colitis score on the indicated day before or post-DSS treatment (n = 8 mice/group). (**d**) Immunofluorescence staining for mucus (MUC2, green), bacteria (FISH, red), nuclei (Dapi, blue), and quantification of MUC2 (**e**) and bacterial distance from intestinal epithelial cells (**f**) in LFD and HFD mice on the indicated day before or post-DSS treatment (n = 4 mic/group). An average measurement of 4 high-powered field (HPF) images per mouse was used for image quantification. The scale bar equals 100 microns (50 microns for the inset). Relative cecal gene expression of (**g**) *Il23a* and (**h**) *Il22* in non-DSS and DSS-treated (day 7) LFD and HFD mice (n = 4 LFD and HFD, n = 5 LFD D7 and n = 9 HFD D7 mice/group). Data are presented as mean ± SEM. *P<0.05, **P<0.001, ***P<0.001, ****P<0.0001. Statistical comparisons were performed using One-way ANOVA with Uncorrected Fisher’s LSD multiple comparisons test; if not indicated, a comparison is not significant.

To assess the influence of HFD feeding on antimicrobial responses after intestinal damage, we used our previous model of feeding male C57BL/6 mice with a 10% low-fat diet (LFD) or 60% high-fat diet (HFD) for one week followed by treatment with 2% DSS in the drinking water for 5 days to induce intestinal injury^2^. Mice placed on LFD or HFD alone had equivalent weight gain (Fig. 1a). After 5 days of DSS exposure, LFD and HFD mice showed similar decreases in body weight and comparable intestinal damage as measured by colitis score (Fig. 1b,c). On day 9, four days post-DSS treatment, improved body weight and resolution of intestinal damage were seen in LFD-fed DSS-treated mice (Figs. 1b,c). However, HFD-feeding in mice exposed to DSS resulted in continued body weight loss and sustained intestinal pathology after DSS treatment (Fig. 1b,c). In agreement with our previous findings, these defects were localized to the cecum and recapitulated our prior observations of HFD-induced defects in intestinal damage repair ^2^.

We previously reported that intestinal healing defects in HFD fed DSS treated mice were associated with decreased mucus production and increased intestinal microbial-epithelial interactions ^2^. By IF staining for the mucus protein mucin 2 (MUC2) and fluorescence in situ hybridization (FISH) to detect microbe-epithelial interactions, we found comparable mucus production and bacterial distance from the intestinal epithelium with both diets alone on day 0 (Fig. 1d–f). Equivalent disruption of the mucus layer was seen in both diet groups on day 5 of DSS treatment, corresponding with similarly increased bacteria-epithelial interactions (Fig. 1d–f). In contrast, on day 9 after DSS treatment, we found decreased mucus and increased microbial-intestinal epithelial interactions in HFD compared to LFD-fed mice (Fig. 1d–f), recapitulating our previous findings ^2^.

Goblet cell mucus production is critical in preventing bacterial-intestinal epithelial cell (IEC) interactions, which further elicit tissue-damaging immune responses and impede damage resolution ^10,11^. Macrophage production of the cytokine IL-23 induces T cell and ILC production of the cytokine IL-22, which induces goblet cell proliferation and mucus secretion^10,12^. We used qPCR to determine whether *Il23a* and *Il22* were normally induced in the cecum of LFD and HFD-fed control and DSS-treated mice. We saw no difference in *Il23a* or *Il22* expression between HFD and LFD-fed mice (Fig. 1g,h). After DSS treatment, cecal *Il23a* and *Il22* expression were significantly induced in LFD-fed mice (Fig. 1g,h). However, *Il23a* and *Il22* responses were severely blunted in DSS-treated HFD-fed mice (Fig. 1g,h). There were no significant differences in the cecal expression of the *Il12a* subunit of IL-12 or the *Il12b* subunit of IL-23 in LFD and HFD-fed mice after DSS treatment (**Supplementary Fig. 1a,b**). These findings suggest that a blunted IL-23 and IL-22 response contributed to decreased mucus production and increased bacterial-epithelial interactions in HFD-fed DSS-treated mice.

### IL-22 overexpression ameliorates intestinal damage repair defects in HFD DSS mice

**Figure 2:**
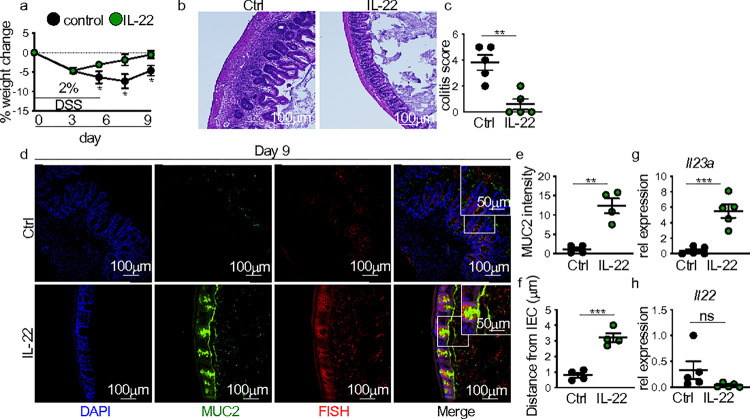
IL-22 overexpression sufficiently attenuates unresolved intestinal damage in HFD mice. (**a**) Body weight of HFD DSS mice with hydrodynamic delivery of control or IL-22 expressing plasmid (n = 5 control n = 8 IL-22 mice/group). The following panels are measurements in the cecum of mice in panel (**a**). (**b**) Representative H&E staining and (**c**) blinded colitis score on the indicated day before or post-DSS treatment (n = 5 mice/group). (**d**) Representative staining for and quantification of (**e**) MUC2 intensity and (**f**) bacterial encroachment (n = 4 mice/group). For all imaging, an average of 4 HPF images were taken per mouse. Relative cecal gene expression of (**g**) *Il23* and (**h**) *Il22* in non-DSS and DSS (day 7) treated LFD and HFD mice (n = 5 mice/group). Data are presented as mean ± SEM. *P<0.05, **P<0.01, ***P<0.001. Statistical comparisons were performed using Studenťs *t* test or One-way ANOVA with Tukey multiple comparisons test and if not indicated, a comparison is not significant. The scale bar equals 100 or 50 microns as indicated.

IL-22 supports mucosal healing by inducing IEC migration needed for wound closure and antimicrobial mucus production ^12–15^. We next determined whether overexpression of IL-22 during the injury recovery phase after DSS treatment could attenuate the repair defects seen in HFD-fed DSS-treated mice. Using a hydrodynamic delivery method, mice were administered a control or IL-22 overexpression plasmid. Overexpression of IL-22 protected HFD mice from increased body weight change and intestinal pathology compared to a control plasmid (Fig. 2a–c). These effects corresponded with increased mucus production and decreased association of microbes with the intestinal epithelium (Fig. 2d–f). While we saw increased cecal *Il23a* expression in HFD-fed DSS-treated mice in response to IL-22 overexpression, we did not see restoration of cecal *Il22* production (Fig. 2g,h). *Il12p40* expression also remained similar between groups, whereas *Il12p35* expression increased (**Supplementary Fig. 2a,b**). Taken together, these data suggest that reduced IL-22 induction in HFD-fed DSS-treated mice contributed to the loss of intestinal mucus production and defective intestinal damage repair.

### Dietary lipids suppress macrophage IL-23 response to intestinal damage and LPS

**Figure 3:**
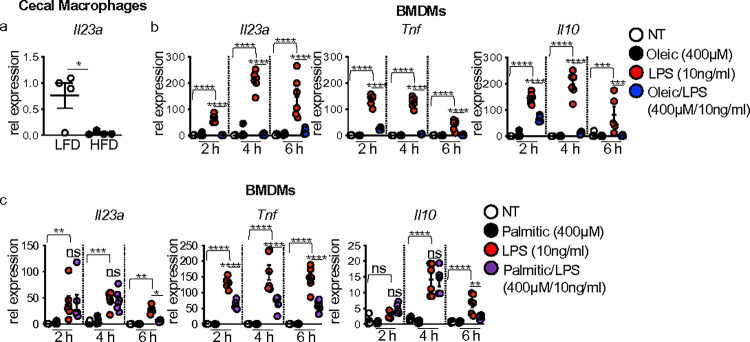
Oleic acid impairs macrophage IL-23 response to intestinal damage and LPS. (**a**) *Il23* gene expression in flow-sorted macrophages from the cecum of LFD and HFD at day 7 post-DSS treatment (n = 4 mice/group). (**b**) *Il23*, *Tnf*, and *Il10*gene expression in BMDMs left un-treated (DMEM) or after treatment with oleic acid (400 μM), LPS (10ng/ml), or oleic acid/LPS (400mm, 10ng/ml) for 2, 4, and 6 h (n = 3 experimental replicates/treatment group, data shown is two experiments). (**c**) *Il23*, *Tnf*, and *Il10* gene expression in BMDMs left un-treated (DMEM) or after treatment with palmitic acid (400 μM), LPS (10 ng/ml), or palmitic acid/LPS (400 μM/10 ng/ml) for 2, 4, and 6 h (n = 3 experimental replicates/treatment group, data shown is two experiments). Data are presented as mean ± SEM. *P<0.05, **P<0.01 ***P<0.001, ****P<0.00001. Statistical comparisons were performed using Studenťs *t* test or One-way ANOVA multiple comparisons with Uncorrected Fisher’s LSD, and if not indicated, a comparison is not significant.

Within the intestine, macrophages serve as one source of IL-23 that drives IL-22 production by innate lymphoid cells and T cells ^4^. We sorted cecal macrophages from mice fed HFD or LFD and treated with DSS as above, and measured *Il23a* gene expression by qPCR. We found significantly reduced *Il23a* expression in cecal macrophages from HFD compared to LFD-fed mice (Fig. 3a). This data paralleled the decreased cecal *Il23a* expression we found in HFD-fed mice above (Fig. 1g).

Dietary lipids are well-known for their direct impact on macrophage function in diet-associated diseases such as obesity and atherosclerosis ^16–19^. We previously demonstrated that the unsaturated lipid oleic acid, comprising 50% of the HFD, impaired macrophage tissue repair functions such as clearance of apoptotic neutrophils, demonstrating this effect as contributing to defective healing of intestinal damage in HFD-fed mice ^2^. We postulated that the oleic acid could also directly influence macrophage IL-23 responses to microbes or microbial signals encountered during intestinal injury.

To investigate this question, we assessed *Il23a* gene expression in bone-marrow-derived macrophages (BMDMs) left untreated, treated with oleic acid alone, LPS alone, or co-treated with oleic acid and LPS for 2, 4, and 6 h. *Il23a* was not expressed in untreated or oleic acid singly treated BMDMs throughout the time course (Fig. 3b). A robust induction of *Il23a* was seen in BMDMs exposed to LPS following 2, 4, and 6 h of treatment (Fig. 3b). *Il23a* expression was severely blunted in oleic acid and LPS co-treated cultures (Fig. 3b). Oleic acid co-exposure had similar repressive effects on *Tnf* and *Il10* expression (Fig. 3b). Dose-dependent effects of oleic acid demonstrated that the suppresses effects on *Il23a*, *Tnf*, and *Il10* were concentration dependent (**Supplementary Fig. 3a-d**). When performing similar assays using the saturated lipid palmitic acid, which comprises ~49% of the lipids in the HFD, we found that palmitic acid alone did not induce *Il23a*, *Tnf*, and *Il10* expression in BMDMs (Fig. 3c). Co-treatment with palmitic acid and LPS resulted in reduced expression of *Tnf* at all time points, and *Il23a* and *Il10* expression were reduced at 6 h post-treatment (Fig. 3c). Suppressive effects of palmitic acid on LPS-induced TNF were concentration dependent (**Supplementary Fig. 4a-d**). These findings demonstrate that lipids in the HFD alter macrophage cytokine expression in response to microbial stimuli and suggest lipid-specific regulation of LPS-induced macrophage expression of *Il23a*, *Tnf*, and *Il10*.

### Macrophage CD36 modulates intestinal repair responses

**Figure 4:**
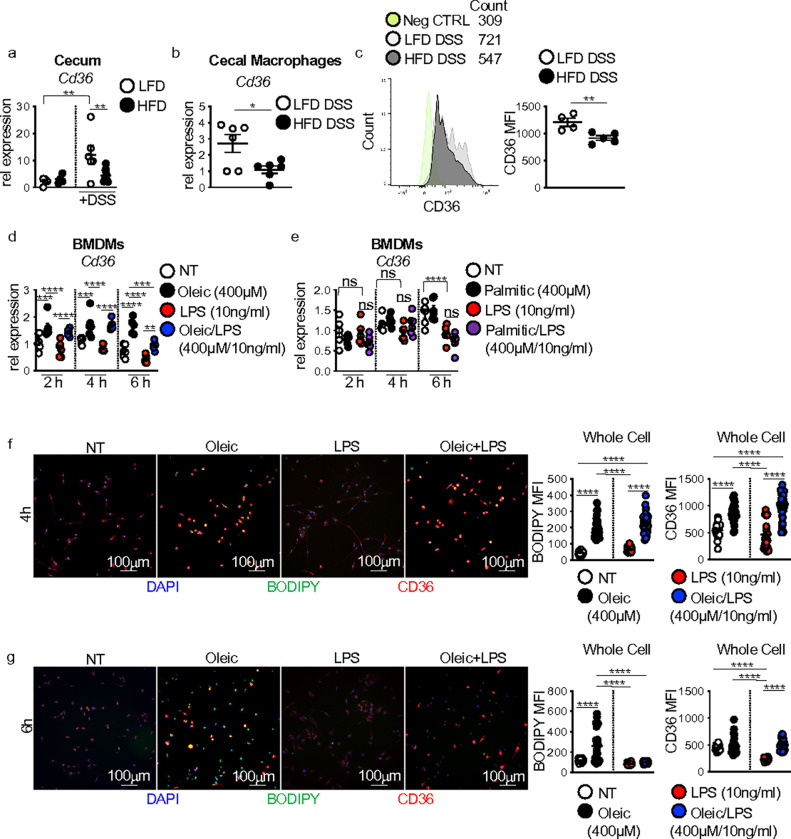
CD36-mediated lipid uptake modulates macrophage antimicrobial response to intestinal damage and LPS. (**a**) CD36 gene expression in the cecum of LFD or HFD mice before or at day 7 post-DSS treatment (n = 4 LFD and HFD, n = 5 LFD DSS and n = 9 HFD DSS mice/group). (**b**) CD36 gene expression (n = 6 mice per group) and (**c**) mean fluorescent intensity (MFI) of CD36 surface level expression in flow-sorted macrophages from the cecum of LFD and HFD mice at day 7 post-DSS treatment (n = 5 mice LFD and n = 4 mice HFD). (**d**) CD36 gene expression in non-treated, oleic acid, LPS, or oleic acid/LPS treated BMDMs at 2, 4, and 6 h (n = 3 experimental replicates/treatment group, data shown represents two experiments). (**e**) CD36 gene expression in non-treated, palmitic acid, LPS, or palmitic acid/LPS treated BMDMs at 2, 4, and 6 h (n = 3 experimental replicates/treatment group, data shown represents two experiments). IF staining for and MFI quantification of CD36 (red) and neutral lipid staining BODIPY (green) in BMDMs exposed to the treatment conditions in (d) for (**f**) 4 or (**g**) 6 h (n = 3 experimental replicates and 2 experiments). For all imaging and quantification, an average of 10 HPF images were taken per sample. Data are presented as mean ± SEM. *P<0.05, **P<0.01, ***P<0.001, ****P<0.0001. Statistical comparisons were performed using One-way ANOVA multiple comparisons with Uncorrected Fisher’s LSD or Tukey’s multiple comparisons, or Studenťs *t* test and if not indicated, a comparison is not significant. The scale bar equals 100 microns as indicated.

Macrophage uptake of lipids through the scavenger receptor CD36 is associated with altered macrophage functions and disease pathogenesis in obesity and atherosclerosis ^16–19^. We observed that diet alone did not alter CD36 expression in the cecum (Fig. 4a). After DSS treatment, CD36 expression increased in the cecum of LFD-fed mice but remained low in the cecum of HFD-fed mice (Fig. 4a). We also found lower gene and surface level protein expression of CD36 in sorted cecal macrophages from HFD compared to LFD-fed mice (Fig. 4b,c).

Using our in vitro lipid and LPS time course assay, we evaluated whether dietary lipid influences on BMDM *Il23a*, *Tnf*, and *Il10* expression corresponded with changes in CD36 expression. Untreated or LPS-alone exposed BMDMs displayed similar levels of CD36 expression (Fig. 4d). Oleic acid exposure alone or with LPS markedly increased CD36 expression at 2 and 4 h, with expression decreasing at 6 h in the oleic acid and LPS co-treated groups (Fig. 4d). Palmitic acid treatment alone or in the presence of LPS resulted in decreased CD36 expression at 6 h post-treatment (Fig. 4e). We next asked how intracellular lipid accumulation corresponded to CD36 expression. We used immunofluorescence to measure the accumulation of the neutral lipid dye BODIPY alongside CD36 expression in oleic acid and oleic acid LPS co-treated BMDMs. At 4h post-treatment, we found accumulation of BODIPY along with increased BMDM expression of CD36 (Fig. 4f). In contrast, at 6 h post-treatment, while we still saw BODIPY accumulation, CD36 expression had decreased (Fig. 4g). These findings suggest a potential link between CD36-mediated macrophage uptake of lipids and lost macrophage cytokine responses to microbial signals.

### Macrophage-specific deletion of CD36 restores the IL-23-IL-22 response in HFD DSS mice

**Figure 5:**
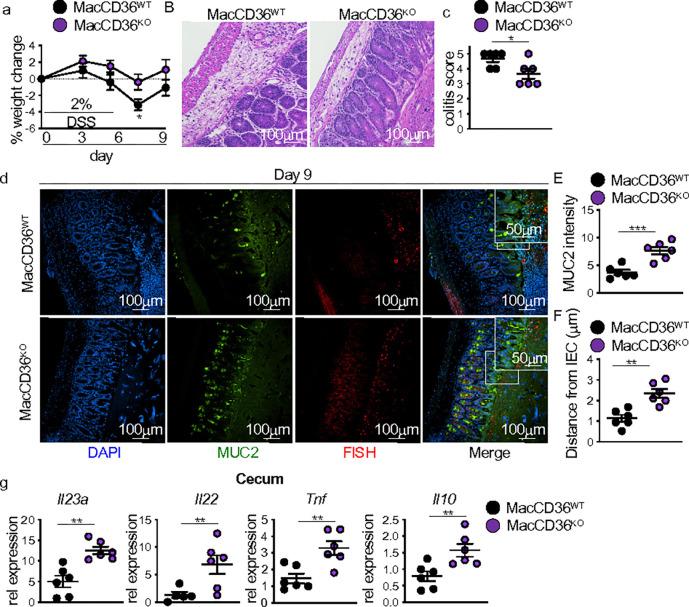
Macrophage-specific loss of CD36 restores reparative antimicrobial responses in HFD DSS mice. (**a**) Body weight (n = 8 MacCD36^WT^ and n = 12 MacCD36^KO^), (**b**) representative H&E, and (**c**) blinded colitis scores of MacCD36^WT^ and MacCD36^KO^ HFD DSS-treated mice (day 9) (n = 6 mice/group). (**d**) Representative staining for and quantification of (**e**) MUC2 intensity and (**f**) bacterial encroachment (n = 6 mice/group). For all imaging, an average of 4 HPF images were taken per mouse. Relative cecal gene expression of *Il23a*, *Il22*, *Tnf*, and *Il10* of mice in MacCD36^WT^ and MacCD36^KO^ HFD DSS-treated mice (day 9) (n = 6 mice/group). Data are presented as mean ± SEM. *P<0.05, **P<0.01, ***P<0.001. Statistical comparisons were performed using Studenťs *t* test, and if not indicated, a comparison is not significant.

We next investigated the role of CD36 in modulating macrophage IL-23-IL-22 responses in HFD mice with intestinal injury. We used HFD feeding and DSS exposure in control (MacCD36^WT^) mice and mice with macrophage-specific deletion of CD36 (MacCD36^KO^) induced by Tamoxifen administration. HFD-fed MacCD36^KO^ mice showed improved body weight change and decreased histopathology compared to WT after DSS treatment (Fig. 5a–c). Improved outcomes corresponded with increased MUC2 levels and decreased bacterial-epithelial interactions in the cecum of HFD MacCD36^KO^ mice compared to MacCD36^WT^ mice (Fig. 5d–f). We next examined cecal *Il23a* and *Il22* expression and found increased gene expression of *Il23* and *Il22* in HFD-fed MacCD36^KO^ compared to WT mice after DSS treatment (Fig. 5g). Furthermore, HFD-fed MacCD36^KO^ DSS-treated mice displayed significantly increased gene expression of *Tnf* and *Il10* (Fig. 5g). These results demonstrated that loss of macrophage CD36 preserves antimicrobial responses to intestinal injury in HFD-fed mice and protects against intestinal damage repair defects seen after HFD feeding.

### The PPARd and BCL6 transcriptional repressor complex governs oleic-acid repression of macrophage IL-23 responses to LPS

The above findings suggest that diminished IL-23-IL-22 responses in HFD DSS mice could result from CD36-mediated macrophage uptake of lipids. CD36 lipid transport and signaling transduction functions, including cytokine production, influence macrophage functions. Next, we sought to identify potential downstream modulators of CD36-mediated oleic acid-induced suppression of macrophage IL-23 and IL-22 responses to intestinal damage in HFD DSS mice. We began our assessment with the intracellular lipid-responsive transcriptional and lipid metabolism regulators, peroxisome proliferator-activator receptors, PPARg and PPARd ^20–22^. PPARg and PPARd can transcriptionally regulate a specific subset of macrophage cytokine responses to microbial stimuli such as LPS ^22^. We postulated that PPAR activity downstream of CD36-mediated oleic acid accumulation in macrophages modulated the IL-23 response to LPS.

**Figure 6:**
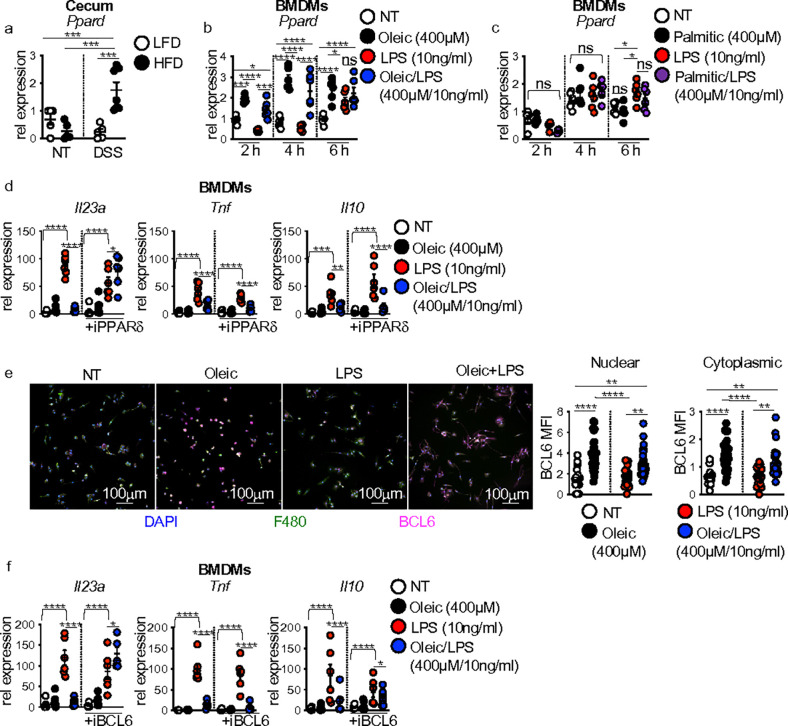
Dietary lipid activation of PPARd and BCL6 suppresses macrophage IL-23 response to LPS. (**a**) Cecal gene expression of *Ppard* in LFD and HFD mice before and after (Day 7) DSS treatment. (**b**) Gene expression of *Ppard*in BMDMs left untreated or treated with oleic acid (400μM), LPS, or oleic acid/LPS (400 μM/10 ng/ml) at 2, 4, and 6 h (n = 3 experimental replicates/treatment group, data shown represents two experiments). (**c**) Gene expression of *Ppard* in BMDMs left untreated or treated with palmitic acid (400 μM), LPS, or palmitic acid/LPS (400 μM / 10 ng/ml) at 2, 4, and 6 h (n = 3 experimental replicates/treatment group, data shown represents two experiments). (**d**) Gene expression of *Il23a*, *Tnf*, and *Il10* in BMDMs left untreated or treated with oleic acid, LPS, or oleic acid/LPS in the presence or absence of PPARd antagonist GSK-3787 (50 μM) for 4 h (n = 3 experimental replicates/treatment group, data shown represents two experiments). (**e**) Representative images of nuclei (DAPI), BCL6 (red) in BMDMs (F480, green), and MFI quantification of nuclear and cytoplasmic BCL6 (n = 3 experimental replicates and 2 experiments). (**f**) Gene expression of *Il23a*, *Tnf*, and *Il10* in BMDMs left untreated or treated with oleic acid, LPS, or oleic acid/LPS in the presence or absence of BCL6 antagonist 79–6 (50 μM) for 46 h (n = 3 experimental replicates/treatment group, data shown represents two experiments). Data are presented as mean ± SEM. *P<0.05, **P<.01, ***P<.001, ****P<.0001. Statistical comparisons were performed using One-way ANOVA with Bonferroni or Uncorrected Fisher’s LSD multiple comparisons, and if not indicated, a comparison is not significant.

First, we assessed cecal expression of *Pparg* and *Ppard* in LFD-fed and HFD-fed mice before and after DSS exposure. Before DSS treatment, cecal *Pparg* and *Ppard* expression were similar between both diet groups (**Supplementary Fig. 5a and Fig. 6a**). In response to DSS treatment, *Pparg* expression was slightly increased but to similar levels in both diet groups (**Supplementary Fig. 5a**). Interestingly, the expression of *Ppard* was significantly induced in HFD-fed compared to LFD-fed mice in response to DSS treatment (Fig. 6a), corresponding with decreased cecal *Il23a* and *Il22* in HFD-fed mice in response to DSS treatment (Fig. 1g,h).

These above results led us to investigate whether the transcriptional response of *Pparg* and *Ppard* in BMDMs exposed to lipids and LPS paralleled changes in cecal *Pparg* and *Ppard* in HFD-fed mice exposed to DSS. In our lipid and LPS time course assay, after 2 h of exposure, BMDM *Pparg* expression slightly increased in oleic acid alone, LPS, and oleic acid/LPS treated groups compared to no treatment but was similarly expressed in all groups at 4 and 6 h post-treatment (**Supplementary Fig. 5b**). When using palmitic acid as the dietary lipid source in these assays, no significant difference in *Pparg* expression was observed throughout the time course (**Supplementary Fig. 5c**). In contrast to *Pparg*, oleic acid treatment alone or with LPS enhanced *Ppard* gene expression in BMDMs at 2 and 4 h compared to no treatment or LPS-alone treatment, with only oleic-alone treatment remaining significant 6 h (Fig. 6b). These oleic acid-induced changes in *Ppard* expression nicely corresponded with increased cecal *Ppard* expression in HFD-fed DSS treated mice (Fig. 6a) and decreased expression of *Il23a*, *Tnf*, and *Il10* in oleic acid/LPS-treated BMDMs (Fig. 3b). *Ppard* expression was not influenced by palmitic acid treatments (Fig. 6c).

As lipid effects on BMDM *Il23a*, *Tnf*, and *Il10* response to LPS were concentration-dependent, we next assessed the dose-dependent responses of *Ppard* and *Pparg* in BMDMs treated with oleic and palmitic acid at 4 h. Oleic acid concentrations of 100 μM or 200 μM had no or a mild impact on *Ppard* and *Pparg* gene expression (**Supplementary Fig. 6a,b**). At 300 μM and 400 μM, oleic acid alone or with LPS significantly increased *Ppard* gene expression compared to no treatment or LPS treatment alone, but had no impact on *Pparg* gene expression (**Supplementary Fig. 6c,d**). Palmitic acid slightly decreased *Ppard* expression at 100 μM in the presence of LPS but did not influence *Pparg* expression at any concentration or *Ppard* expression at 200 μM and above (**Supplementary Fig. 7a,d**). Collectively, these findings demonstrated that *Ppard* induction by high concentrations of oleic acid corresponded with oleic acid inhibition of BMDM *Il23a*, *Tnf*, and *Il10* response to LPS.

To gain insight into whether oleic acid induction of *Ppard* translated to PPARd repression of *Il23a*, *Tnf*, and *Il10* in BMDMs in response to LPS, we co-exposed lipid and LPS-treated BMDMs with or without the potent irreversible small molecule PPARd inhibitor GSK-3787 ^23^. As expected, oleic acid suppressed LPS-induced *Il23a*, *Tnf*, and *Il10* gene expression levels compared to LPS treatment alone (Fig. 6d). Interestingly, inhibition of PPARd restored BMDM *Il23a* expression response to LPS but not expression of *Tnf* or *Il10* (Fig. 6d). These data demonstrate that PPARd governs oleic acid repression of macrophage *Il23a* expression response to microbial signals in vitro and suggest an alternate regulatory mechanism modulates oleic acid suppression of *Tnf* and *Il10* in macrophages. Furthermore, these data imply that increased PPARd activity in macrophages could contribute to the loss of IL-23 and IL-22 signaling in HFD mice with intestinal damage.

PPARd influences on macrophage cytokine responses can be mediated by the transcriptional repressor B cell lymphoma 6 (BCL6) ^24^. Liganded PPARd releases BCL6 to repress NFkB target genes ^24,25^, such as IL-23 ^26^. We first used immunofluorescence staining to assess BCL6 expression levels in non-treated, oleic acid alone, LPS alone, or oleic acid and LPS co-exposed BMDMs. BCL6 expression levels assessed by mean fluorescence intensity (MFI) demonstrated that oleic acid treatment alone or with LPS significantly increased BCL6 protein levels in BMDMs compared to no treatment or LPS (Fig. 6e). To determine the contribution of BCL6 in oleic acid repression of macrophage cytokine response to LPS, we co-exposed untreated, oleic acid alone, LPS alone, or oleic acid/LPS-treated BMDMs with the BCL6 small molecule inhibitor 79–6 ^27^. As with antagonism of PPARd, inhibition of BCL6 restored macrophage *Il23a*, but not *Tnf* and *Il10*, response to LPS (Fig. 6f). These findings demonstrate that oleic acid modulation of BCL6 activity through PPARd represses macrophage IL-23 response to LPS and highlight direct lipid-specific regulation of macrophage antimicrobial IL-23 response.

## Discussion

High animal fat diets are a risk factor for IBD, and the excessive lipid content of the HFD is well-known to directly alter immune functions to support the development or pathogenesis of diet-associated diseases such as obesity and atherosclerosis ^28–34^. Yet, whether the direct influences of HFD lipids on intestinal immune reparative functions also contribute to the pathogenesis of IBD is less understood. Our findings provide evidence that direct effects of HFD lipids on macrophage antimicrobial and tissue reparative functions support defects in intestinal damage repair, which could further exacerbate IBD pathogenesis. Previous reports demonstrate a link between HFD-induced and genetic obesity and the loss of the IL-23-IL-22 responses in an infectious model of colitis using *Citrobacter rodentium* infection in mice ^35^. In these studies, obese mice were unable to clear *Citrobacter* infection, where pathogen clearance is dependent on IL-23 and IL-22 signaling, and sustained more intestinal damage. As with our studies, administration of IL-22 resolved intestinal damage after epithelial damage and pathogen infection. Although not investigated in these studies, the lack of IL-22 responses to *Citrobacter* infection in the context of HFD-induced obesity was suggested to be due to the loss of dendritic cell IL-23. Our findings show that reduced macrophage IL-23 response to intestinal injury after HFD feeding also underlies unresolved damage in mice with intestinal injury. These previously published findings and our current study highlight how dietary fats govern critical innate immune responses, notably IL-23 and IL-22 responses, to intestinal injury or pathogen infection and dictate the hosťs ability to resolve intestinal injury. Identification of additional macrophage reparative functions modulated by the lipids could further increase our understanding of the connection between HFD consumption and IBD risk.

Our ex vivo and in vitro studies provide evidence that specific lipids in the HFD directly repress macrophage antimicrobial and reparative cytokine responses to intestinal damage. Our findings reveal that the intracellular presence of the dietary lipid oleic acid in BMDMs drove repressed *Il23a*, *Tnf*, and *Il10* responses to LPS, mirroring our in vivo findings. Oleic acid, an unsaturated fatty acid, produces an anti-inflammatory phenotype in macrophages ^36–38^. In general, the anti-inflammatory effects of oleic acid are suggested to benefit some aspects of IBD, such as dampening inflammatory responses ^39–41^. However, our studies indicate that although oleic acid may have beneficial anti-inflammatory effects, these same responses interfere with protective functions, like IL-23 production, downstream of microbial signals needed to support the healing of the intestinal epithelial lining, which may further exacerbate intestinal damage. Interestingly, the saturated lipid palmitic acid, which is associated with an inflammatory macrophage phenotype and IBD ^38,41,42^, only directly influenced macrophage *Tnf* responses to LPS and not cytokines such as *Il23a* and *Il10* that influence repair. As dietary fat association with altered IBD risk is complex, continued study is needed to understand how dietary lipid components influence various intestinal cell types, intestinal repair process, damage, and inflammation in the gut. Specific to our studies, future use of single-source lipid-enriched diets compared to complex combinations of lipids is needed to understand the complex influence of specific lipid components and combinations of lipids on macrophage antimicrobial reparative responses in IBD.

CD36 modulation of the intracellular lipid content in macrophages directly influences the pathogenesis of atherosclerosis and obesity ^18,43,44^. Our in vivo studies suggest that macrophage CD36 similarly contributes to the pathogenesis of IBD by facilitating the transport and accumulation of diet-derived lipids in macrophages, altering macrophage responses to microbial signals. The restoration of *Il23a*, *Il22*, *Tnf*, and *Il10* we see in mice with macrophage-specific CD36 deletion suggests that CD36-mediated lipid transport and signaling transduction play critical roles in intestinal immune microbial defense and repair response. In the intestine, there is evidence that CD36 plays a role in intestinal barrier function, as global loss of CD36 in mice impairs the small intestinal barrier and induces subclinical inflammation under normal dietary conditions ^45^. More recent studies demonstrate a role for CD36-mediated transport of long-chain fatty acids by intestinal epithelial cells in driving colonic inflammation ^42^. Collectively, these findings highlight the important role of CD36 in modulating inflammatory and repair responses in the intestine. How CD36 influences the function of various intestinal cell types and the impact on intestinal disease pathogenesis should be further studied.

PPARd serves as an intracellular lipid sensor that modulates lipid-induced inflammatory responses in macrophages ^20,25,33^. Our in vitro studies reveal that increased macrophage PPARd activity induced by intracellular oleic acid accumulation precludes LPS-induced macrophage production of IL-23. Limiting PPARd activity attenuated the loss of macrophage IL-23 response to LPS. This phenotype mirrored our in vivo findings of lost cecal macrophage IL-23, suggesting that excessive PPARd activity drives lost macrophage reparative antimicrobial responses and perpetuated defects in intestinal healing. PPARd effects on macrophage inflammatory response are mediated through the transcriptional repressor BCL6 ^24^. Lipid-liganded PPARd releases BCL6 to repress many NFkB target genes ^24^. With relevance to the antimicrobial response needed to promote intestinal damage, BCL6 deficiency in macrophages increases LPS-induced IL-23 production and IL-22 production from TH17 cells ^26^. Our studies show that BCL6 transcriptional repressive activities downstream of lipid activation of PPARd suppress macrophage IL-23 induction after LPS treatment. Targeting BCL6 in T cells is suggested as a potential druggable target for IBD due to increased BCL6 expression in this population correlating with IBD pathogenesis and specific IBD subtypes ^46–48^. Our studies suggest that modulation of macrophage BCL6 may be beneficial to improve intestinal immune damage repair responses. More in-depth studies will help define the role of BCL6 in modulating intestinal healing. Developing strategies to regulate the PPARd and BCL6 activity to promote the reparative effects of IL-23 and IL-22 may be a novel treatment strategy for restoring barrier integrity and slow diet-driven IBD occurrence and progression.

## Materials and Methods

### Experimental Animals

All animal experiments were performed with approved protocols by the Institutional Animal Care and Usage Committee at Baylor College of Medicine and Memorial Sloan Kettering Cancer Center. All animal research reported in this paper is in accordance with ARRIVE guidelines ^49^. Animals used in this study were C57BL/6J (JAX #000664), CX_3_CR1^+^ GFP/+ (JAX # 005582), CX_3_CR1-CreERT2 (JAX# 021160), and CD36flox tm1.1Ijg/J (JAX # 032276) purchased from Jackson Labs. Littermate controls were used for each experiment and mice were randomly assigned to experimental groups. Animals were housed under standard specific pathogen-free (SPF) conditions at Baylor College of Medicine or Memorial Sloan Kettering Cancer Center animal facility. All lines were backcrossed for at least 12 generations to the C57BL/6J background. To generate MacCD36^ko^ mice, CD36^fl/fl^ mice were crossed with CX_3_CR1CreERT2, and mice (CX_3_CR1CreERT2 and control) were injected i.p. with 0.2mg with (Z)-4-Hydroxytamoxifen, 98% Z isomer (4OHT, Sigma) every 3 days starting on Day 0 of DSS treatments. Tamoxifen was resuspended to 20mg/ml in ETOH with heating to 37°C. 40HT was diluted to 0.2mg in a 100ul corn oil (Sigma). At least 4 mice per group between 6 and 8 weeks of age were used for all mouse experiments. Multiple combined experiments were used to assess statistical significance.

### Acute diet feeding and intestinal injury

Mice were fed 10% kcal low fat diet (LFD) (Research Diets, D12450B) or 60% kcal high-fat diet (HFD) (Research Diets, D12492) ad libitium for one week prior to treatment with 2% Dextran sodium sulfate (DSS, ThermoFisher, AAJ1448922) in drinking water for 5 days followed by plain drinking water.

### Histology

Mouse cecums were fixed in Carnoy’s fixation for 1–2 days before being placed in methanol prior to paraffin embedding. Samples were deparaffinized, cut into 4μM sections, and stained with hematoxylin. Images were taken with a Nikon Ti Eclipse microscope. Sections from 4–6 mice were used for blinded colitis scoring according to established criteria ^50,51^ and as previously described ^2^.

### Immunofluorescence tissue staining

Before antibody IF staining, Fluorescence In Situ Hybridization (FISH) staining was performed prior to immunofluorescence staining as described in^2,52^ using the following UNI519 universal primer-probe sequence: /5Alex594N/GTATTACCGCGGCTGCTG (Integrated DNA Technologies (IDT). Sections were washed 1x with PBS and 1x with MilliQ water. Sections were permeabilized, blocked, and stained overnight at 4°C with the following primary antibodies at a 1:100 dilution: MUC2 (polyclonal, Cloud Clone Cat# PAA705Mu02). After 2X wash with 1x TBST and 1X wash with 1x PBS, sections were stained with a 1:200 dilution of the secondary antibody anti-rabbit Alexa 488 (Cell signaling Cat# 4412) for 1 h. Sections were washed 1X with 1x PBS and stained with DAPI (Sigma Aldrich Cat# D9542) and mounted using Aqua Mount (Polysciences Cat# 18606-100) anti-fade mounting media and coverslipped. Images were taken on Nikon Ti Eclipse microscope using 20x and 40x objectives, and images were processed using FIJI.

### Quantification of immunofluorescence staining

#### MUC2 and FISH.

Three images from 4 mice per group were used to quantify MUC2 intensity and bacterial encroachment. For MUC2 intensity, Image J was used to set a threshold and mask for each image, and pixel intensity was measured using the Image J measuring tool. Bacterial encroachment was measured as the distance between the closest bacteria to the intestinal epithelium using the Image J measuring tool.

### Gene expression

RNA from whole cecum (0.5 in.), sorted cecal macrophages, or cultured BMDMs was isolated using Trizol (Invitrogen) according to the manufacturer’s instructions. iScript reverse transcription kit (Bio-rad Laboratories) was used to synthesized cDNA. Real-time quantitative qPCR was performed using SYBR Green Supermix (Bio-rad Laboratories) using a CFX384 Touch real-time PCR machine. Thermocycling program was 95°C for 2 min followed by 40 cycles at 95°C for 15 s, 60°C for 30s, and 72°C for 30s. The following primers were used: mouse Il23a-F: CCAGCAGCTCTCTCGGAATCT, mouse Il23a-R: AAGCAGAACTGGCTGTTGTC, mouse Il22-F: CATGCAGGAGGTGGTACCTT, mouse Il22-R: CAGACGCAAGCATTTCTCAG mouse Il10-F: CCAGCTGGACAACATACTGCT, mouse Il10-R: AACCCCACAAGAGTTCTTTCAAA, mouse Gapdh-F: AATGTGTCCGTCGTGGATCT, mouse Gapdh: CATCGAAGGTGGAAGAGTGG, mouse Tnf-F: AGGGTCTGGGCCATAGAACT, mouse Tnf-R: CCACCACGCTCTTCTGTCTAC, mouse Cd36-F: AACACTGTGATTGTACCTG, mouse Cd36-R: TCAATAAGCATGTCTCCGAC, mouse Pparg F: GCATGGTGCCTTCGCTGA, mouse Pparg-R: TGGCATCTCTGTGTCAACCATG, mouse Ppard-F: TTGAGCCCAAGTTCGAGTTTG, mouse Ppard-R: CGGTCTCCACACAGAATGATG. Relative expression of target gene was determined using the delta delta CT method. Gapdh was used as an internal control.

### Overexpression of IL-22

One day after the start of DSS treatment, mice were administered a Plasmid DNA control or IL-22 overexpression plasmid (InVivoGen) intravenously (i.v.) at 10μg DNA/mouse diluted in TransIT-EE Hydrodynamic Delivery solution (Mirus) at 0.1 ml/g body weight 1 day after the start of DSS treatment ^4,53^

### Lamina propria cell isolation

Isolation of lamina propria cells was performed as previously described ^2,54,55^. In short, after the luminal contents were removed, the sections were treated with 1mM DTT and 30mM EDTA, followed by 30mM EDTA, both for 10min at 37 degrees to remove mucus and epithelial cells. Tissues were digested in 200U/ml collagenase 8 (Sigma-Aldrich C-2139) and 150μg/ml DNase (Sigma DN25) in RPMI supplemented with 10% FBS while shaking at 37°C for 1 h. Lamina propria cells were isolated using a 40%/80% Percoll (Sigma Aldrich) gradient.

### Flow cytometry and FACS sorting

The following antibodies were used for flow staining and or sorting from CX_3_CR1^+^ GFP/+ (JAX # 005582) mice: MHC II (M5/114.15.2, BioLegend Cat# 107620), CD11b (M1/70, Biolegend Cat# 101226), CD11c (N418, Biolegend, Cat# 117317), Ly6C (AL-21, BD PharMingen Cat#560525), CD45 (30-F11, Biolegend Cat#103149), DAPI (Sigma-Aldrich Cat# D9542). Monocyte-derived macrophages were defined as CX_3_CR1^hi^ CD11b^+^ MHCII^+^ Ly6c^neg^. CD36 (HM36, Biolegend CA#102606) was used to assess CD36 mean fluorescence intensity in cecal macrophages by flow cytometry. Flow cytometry and analysis were performed with an LSR II (BD) and FlowJo software (Tree Star). Dead cells were excluded using the Live/Dead fixable aqua dead cell stain kit (Invitrogen). Macrophages were sorted on a FACSAria Cell sorter (BD Biosciences).

### Bone marrow-derived macrophages (BMDMs)

Bone marrow cells were collected from 6 to 8-week-old male and female C57BL/6 male and female mice and differentiated into BMDMs by culturing in BMDM media for 6 days as previously described ^2,56^. BMDM media: 50% DMEM (Corning) supplemented with 20% FBS, 30% L cell (ATCC CRL-2648) media, 2mM glutamine, 1 mM pyruvate, 1 unit/ml pen/strep, and 55μM β-ME. BMDM media was supplemented every 3 days. Confirmation of macrophage differentiation was assessed by IF staining using the murine macrophage marker F480 (Abcam ab6640). All assays were performed in DMEM supplemented with 10% FBS, 1 unit/ml pen/strep, and 1 mM HEPES.

### LPS and lipid treatments of BMDMs and BODIPY staining

Fatty acids were dissolved in ethanol as described ^30^. BMDMs were treated with 100, 200, 300, or 400μm oleic acid or palmitic acid (Nu-Chek Prep) or an equivalent amount of solvent (ethanol) alone or co-treated with 10ng/ml LPS (Millipore Sigma, L4391) for 2, 4, or 6 h. BMDMs were either collected for gene expression analysis, or stained with primary antibodies: DAPI (Invitrogen D1306), F480 (Abcam ab6640), CD36 (abcam ab252922), BCL6 (Invitrogen PA5-27390); secondary antibodies: Cell Signaling anti-rat Alexa 488 (4416), anti-rat Alexa 647 (4414), R&D systems NorthernLights: NL-555 anti-rabbit (NL007), NL-637 anti-rabbit (NL005), NL-493 anti-rabbit (NL009); and 1um of of the neutral lipid stain BODIPY 493/503 (Invitrogen, D3922) for 30 at RT after immunostaining to assess lipid uptake.

#### Automated Cell Counting.

Microscopy images were processed with Fiji/ImageJ v.2.3.0/1.53f using a custom macro to quantify fluorescence in individual cells ^57^. Briefly, images underwent background correction using the rolling-ball algorithm. Cell boundaries and nuclei were identified to create separate masks: the cell mask was generated using the F480 fluorescence channel, the nuclei mask was generated using the DAPI signal, and the cytoplasmic mask was derived by subtracting the nuclei mask from the cell mask. Fluorescence thresholds for each marker were set manually based on representative images from oleic-treated wells. Thresholds were set separately for two sets of measurements: (1) green (BODIPY), red (CD36), blue (DAPI), and magenta (F480) channels for measuring sample with CD36 MFI and BODIPY, and (2) green (BODIPY), red (F480), blue (DAPI), and magenta (BCL6) channels for measuring sample with BCL6 MFI. For each mask (cell, nucleus, cytoplasm), individual area measurements and the average fluorescence intensities for CD36, BODIPY, and BCL6 were recorded. The number of cells per image was estimated by counting individual DAPI-stained nuclei. Finally, an R script summarized fluorescence intensities for each mask and computed the mean fluorescence per cell by dividing the total fluorescence intensity by the estimated cell number. Analyses were performed using GraphPad Prism version 10.0. The ImageJ macro is available online at: https://bitbucket.org/the-samuel-lab/mcalester-2022/.

### PPARd and BCL6 antagonist treatments

BMDMs were treated with or without LPS and oleic acid as above, in the presence of 50um PPARd antagonist GSK-3787(Abcam ab144575) or BCL6 small molecule inhibitor 79 – 6 (Sigma197345) for 4 h. BMDMs were then collected for gene expression analysis.

### Statistical analysis

One-way analysis of variance (ANOVA) with Tukey’s posttest or unpaired Studenťs t test was performed using a 95% confidence interval. All data are presented as mean ± SEM. All analyses were performed using GraphPad Prism version 10.0. Differences were considered to be significant at P values of less than 0.05.

## Data Availability

The datasets used and/or analyzed during current study are available from the corresponding author upon reasonable request.
